# Suppression of *In Vivo* Neovascularization by the Loss of TRPV1 in Mouse Cornea

**DOI:** 10.1155/2015/706404

**Published:** 2015-09-27

**Authors:** Katsuo Tomoyose, Yuka Okada, Takayoshi Sumioka, Masayasu Miyajima, Kathleen C. Flanders, Kumi Shirai, Tomoya Morii, Peter S. Reinach, Osamu Yamanaka, Shizuya Saika

**Affiliations:** ^1^Department of Ophthalmology, Wakayama Medical University, 811-1 Kimiidera, Wakayama 641-0012, Japan; ^2^Laboratory Animal Center, Wakayama Medical University, 811-1 Kimiidera, Wakayama 641-0012, Japan; ^3^Laboratory of Cell Regulation and Carcinogenesis, National Cancer Institute, National Institutes of Health, Bethesda, MD, USA; ^4^Wenzhou Medical University School of Ophthalmology and Optometry, Wenzhou, China

## Abstract

To investigate the effects of loss of transient receptor potential vanilloid receptor 1 (TRPV1) on the development of neovascularization in corneal stroma in mice. Blocking TRPV1 receptor did not affect VEGF-dependent neovascularization in cell culture. Lacking TRPV1 inhibited neovascularization in corneal stroma following cauterization. Immunohistochemistry showed that immunoreactivity for active form of TGF*β*1 and VEGF was detected in subepithelial stroma at the site of cauterization in both genotypes of mice, but the immunoreactivity seemed less marked in mice lacking TRPV1. mRNA expression of VEGF and TGF*β*1 in a mouse cornea was suppressed by the loss of TRPV1. TRPV1 gene ablation did not affect invasion of neutrophils and macrophage in a cauterized mouse cornea. Blocking TRPV1 signal does not affect angiogenic effects by HUVECs* in vitro*. TRPV1 signal is, however, involved in expression of angiogenic growth factors in a cauterized mouse cornea and is required for neovascularization in the corneal stroma* in vivo*.

## 1. Introduction

The cornea is a unique ocular tissue of avascularity and transparency for proper vision. It is susceptible to neovascularization-inducing intervention, that is, microbial infection or ocular surface damage. Such unfavorable neovascularization potentially impairs vision. The process of the new vessel formation in an injured cornea is orchestrated by a complex system of various growth factor signaling [1–5]. Resident corneal cells and inflammatory cells invaded to an injured tissue express growth factors and cytokines involved in injury-induced neovascularization. Such factors include vascular endothelial growth factor (VEGF), transforming growth factor *β* (TGF*β*), and fibroblast growth factor (FGF) [6–10].

Members of the transient receptor potential (TRP) channel superfamily are polymodal receptors that are activated by a host of stimuli to mediate sensory transduction. The family is divided into 7 different subfamilies and composed of 28 different genes [11–14]. TRP vanilloid subtype 1 (TRPV1), the capsaicin receptor, is a nociceptor and one of the prototypes of TRPV subfamily. It elicits responses to a variety of noxious stimuli including chemical irritants besides capsaicin, inflammatory mediators, tissue injury, an alteration in pH, and moderate heat (≥43°C). TRPV1 activation leads to nociception and evokes pain or pain-related behaviors and reportedly induces release of tachykinin neuropeptides from sensory nerves, inducing neurogenic inflammation in the surrounding area [15–17]. Various nonneuronal cell linages, that is, epidermal keratinocyte or corneal epithelium and keratocytes, also express TRPV1, which presumably exerts a variety of biological responses to external stimuli [18–24]. We previously reported that lacking TRPV1 counteracted inflammatory and fibrogenic reactions in corneal stroma following an alkali burn [24]. The phenotype of less-inflammation/fibrosis was found to depend on the loss of keratocytes in the affected stroma, but not on inflammatory cells as revealed by reciprocal bone marrow transplantation experiments. Stromal neovascularization is also a component of the biological reaction observed in an injured cornea. In our previous study on an alkali-burned cornea, however, we failed to extract the effects of the loss of TRPV1 on injury-induced neovascularization in an alkali-burned cornea due to complex tissue reaction in the stroma. It was reported that capsiate and piperine, both TRPV1 agonists, suppress angiogenic behaviors of vascular endothelial cells cultured in the absence of inflammatory components* in vitro* [25, 26].* In vivo* role of TRPV1 signal in modulation of neovascularization is to be assessed in* in vivo* condition. In the present study we examined the roles of TRPV1 signal in the activity of neovascularization development by using TRPV1-null (KO) mice and* in vitro* human umbilical vein endothelial cell (HUVEC) culture model of neovascularization.

## 2. Materials and Methods

*In vivo* experiments were approved by the DNA Recombination Experiment Committee and the Animal Care and Use Committee of Wakayama Medical University and performed in accordance with the Association for Research in Vision and Ophthalmology Statement for the Use of Animals in Ophthalmic and Vision Research.

### 2.1. Coculture Experiment of Tube-Like Structure Formation by HUVECs

We first employed* in vitro* assay of angiogenic activity of HUVECs. The degree of tube-like structure formation by HUVECs on a fibroblast feeder layer was employed to assess the effects of each agent on neovascularization activity of the cells. The detailed procedure was reported in our previous publications [8, 27]. HUVECs were seeded on the fibroblast feeder layer as the manufacture suggested (NV kit, Kurabo, Tokyo, Japan). Then the culture was maintained in the routine culture condition in the presence of vascular endothelial cell growth factor- (VEGF-) A (10.0 ng/ml, Kurabo, Tokyo, Japan) as an angiogenesis inducer in the presence or absence of a TRPV1 antagonist, SB366791 (10 *μ*M, Sigma-Aldrich). HUVECs did not develop tube-like structure in the absence of VEGF (data not shown). Eight wells were prepared for each culture condition. After 11 days of culture the cells were processed for immunohistochemistry for CD31 (a marker for vascular endothelium) according to the manufacture's protocol. Color development was performed by 3,3′-diaminobentidine (DAB) reaction [8, 27]. Average length and the average number of bifurcations (the number of branching points) were counted in three central fields in each well in a blinded fashion by an investigator. The mean value of the data from these three fields represented the data of each well. Statistical analysis of the data from eight wells was conducted by employing Tukey-Kramer test and *p* < 0.05 was taken as significant.

### 2.2. Induction of Stromal Neovascularization by Cauterization of the Central Cornea in Mice

We then performed an* in vivo* neovascularization assessment experiment by using a wild-type (WT) of C57Bl/6 (*n* = 52) or KO mouse of C57Bl/6 background (*n* = 65) as previously reported [8]. KO mice were healthy without any obvious general abnormalities and were fertile. There was no difference in the histological findings in an uninjured cornea between a WT and KO mice as previously reported [24]. Corneal stromal neovascularization from the limbal vessels was induced by cauterization of the central cornea of an eye of both WT and KO mice by disposable cauterization tool as previously reported [8].

We first observed the morphology of neovascularization at day 3 after cauterization in whole-mounted specimens by using CD34 immunostaining. Four WT and 4 KO mice were used. Mice were sacrificed at day 3 after cauterization in the central cornea by CO_2_ asphyxia. The eye was enucleated, processed for whole-mounted immunostaining. The eyes were fixed in 4% paraformaldehyde for 48 hrs. After washing in phosphate-buffered saline (PBS), the specimens were treated in 0.5% Triton X for 1 hr to facilitate the antibody penetration into the tissue. After rinsing in PBS, the samples were allowed to react with a monoclonal anti-CD31 antibody (1 : 100 in PBS, Santa Cruz Biotechnology Inc., Santa Cruz, CA, USA) for 24 hrs at 4°C. After washing the antibody the tissues were then treated with a FITC-labeled secondary antibody (Southern Biotechnology, Birmingham, Alabama, USA) for 12 hrs at 4°C, mounted in Fluoromount-G after another wash in PBS, and observed under Carl Zeiss Apotome. 2 AxioVision 4.8 fluorescence microscopy.

We then examined the length of neovascularization from limbus toward the center of the cornea following cauterization in histological section. For this purpose, 15, 16, or 8 WT mice and 21, 21, or 10 KO mice were used for assessment at day 3, 7, or 14, respectively. Mice were sacrificed at days 3, 7, and 14 after cauterization in the central cornea by CO_2_ asphyxia. The eye was enucleated, processed for cryosections, and used for immunohistochemistry for CD31 (monoclonal, 1 : 100 in PBS, Santa Cruz Biotechnology Inc.) as previously reported [28]. The length of corneal stromal neovascularization was measured as follows: length between limbus and the tip of neovascularization was measured in both sides of the limbus in three cryosections produced from one eye. The average value of the six data represented the neovascularization in one cornea. Statistical analysis was conducted with the use of Mann-Whitney *U* test, and* p* < 0.05 was taken as significant.

### 2.3. Immunohistochemistry

Cornea of three eyes of each genotype of mice was also cauterized and then processed for paraffin section immunohistochemistry for active form of TGF*β*1, VEGF, substance P, and F4/80 macrophage antigen as previously reported. As described below we semiquantified mRNA expression of TGF*β*1 in treated corneas and TGF*β*1 exerts its action after processing to the active form. We therefore used an antibody that reacts the active, but not inactive, form of TGF*β*1 in the current study [24, 29].

### 2.4. Gene Expression Analysis

We examined the expression of mRNAs of neovascularization-related growth factors and the degree of inflammation in* in vivo* mouse cornea. We considered mRNA level quantification was essential because our preliminary immunohistochemistry for VEGF and TGF*β*1 showed very faint staining with minimal difference of the staining in central corneal stroma between a WT and a KO mouse. Centrally cauterized cornea (*n* = 6 in each of WT or KO group) was excised at day 3. Total RNA was extracted from these tissues and processed for TaqMan real-time reverse transcription-polymerase chain reaction (RT-PCR) for VEGF, TGF*β*1, myeloperoxidase (MPO) and F4/80 macrophage antigen and as previously reported [24]. Delta/delta CT method by Applied Biosystem Inc. was employed with the internal control of glyceraldehyde 3-phosphate dehydrogenase (GAPDH) expression. Primers (Applied Biosystem Inc.) used were described in the following list. Data were statistically analyzed by employing Mann-Whitney *U* test.

*Primers Used (Applied Biosystem Inc.).* Consider

 vascular endothelial growth factor: Mm01281447_ml;
 transforming growth factor *β*1: Mm03024053_ml;
 substance P: Mm01166996_ml;
 interleukin-6: Mm01210732_gl;
 myeloperoxidase: Mm01298422_gl;
 F4/80: Mm00802524_ml.

## 3. Results

### 3.1. *In Vitro *Experiment of Tube-Like Structure Formation by HUVECs

**Figure 1:**
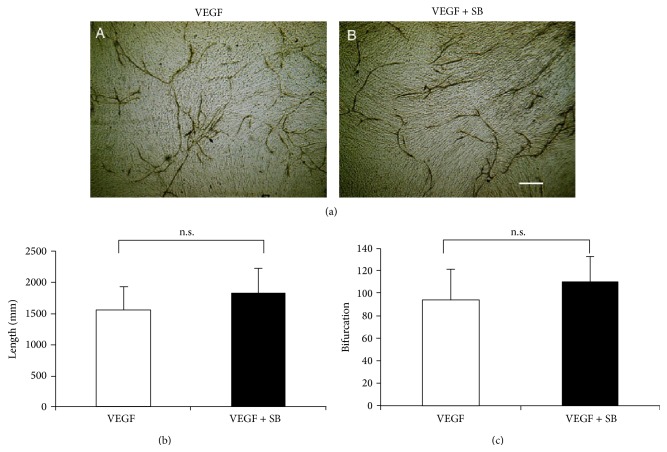
Tube-like structure formation by human umbilical vein endothelial cells (HUVECs). (a) The HUVEC culture on fibroblast feeder was processed for CD31 immunocytochemistry at day 11. In control vascular endothelial growth factor- (VEGF-) plus, culture HUVEC forms CD31-labeled tube-like tissue (A). VEGF action of tube-like structure formation is not affected by supplementation of a TRPV1 antagonist, SB366791 (10 *μ*M) (B). Measurement of total length (b) and the number of branching points (c) of CD31-labeled structure coincide with the findings shown in frame (a). ^∗^ *p* < 0.05, bar, 1 mm.

Dense CD31 immunoreactivity was detected in tissue where HUVECs formed a vessel-like tube structure. Without exogenous VEGF, HUVECs grown on the fibroblast feeder layer did not form a vessel-like tube tissue. The HUVEC culture was processed for CD31 immunocytochemistry at day 11 (Figure 1(a)). The angiogenic behaviors of HUVECs were evaluated by measurement of the mean total length of the structure (Figure 1(b)) and of the men number of bifurcations (Figure 1(c)) in randomly selected fields of the culture as described above. In the culture with VEGF-A (10 *μ*g/ml) CD31-labeled tube-like structure was observed. Supplementation of a TRPV1 antagonist, SB366791 (10 *μ*M), did not affect VEGF-A action on tube-like structure formation by HUVECs (Figure 1).

### 3.2. Neovascularization in Corneal Stroma

**Figure 2:**
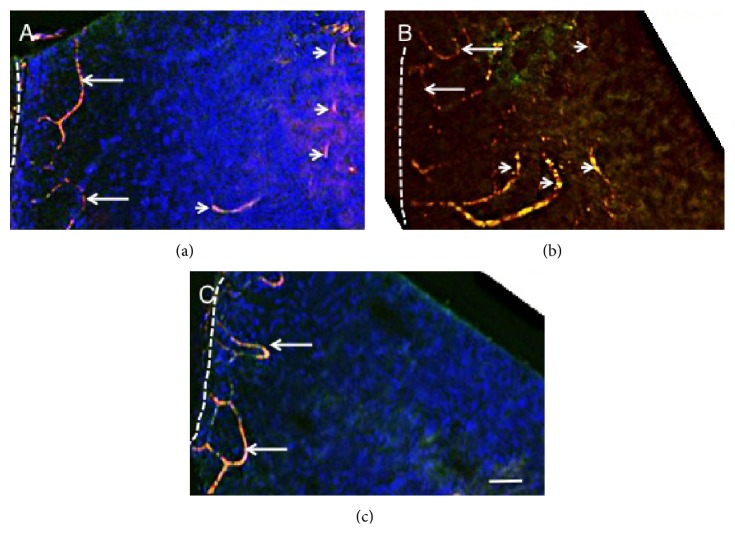
Neovascularization in corneal stroma as observed in whole-mounted specimens. We first observed the morphology of neovascularization in whole-mounted specimens by employing CD34 immunostaining. WT ((a), (b)) and KO (c) corneas at day 3 show loop-like distribution of blood limbal vasculature (arrows in (a), (b), and (c)). In WT corneas neovascularization was observed in the stroma apart from the limbus (arrowheads in (a) and (b)), although the staining procedure did not figure the continuous elongation of the vessels. Dotted lines, limbal corneoscleral border; bar, 100 *μ*m.

We first observed the morphology of neovascularization in whole-mounted specimens by employing CD31 immunostaining.  Figure 2 shows the morphology of limbal vasculature of WT and KO corneas at day 3. In both WT (Figures 2(a) and 2(b)) and KO (Figure 2(c)) corneas loop-like distributions of blood vessels were observed. In WT corneas neovascularization was observed in the stroma apart from the limbus, although the staining procedure did not figure the continuous elongation of the vessels (Figures 2(a) and 2(b)).

**Figure 3:**
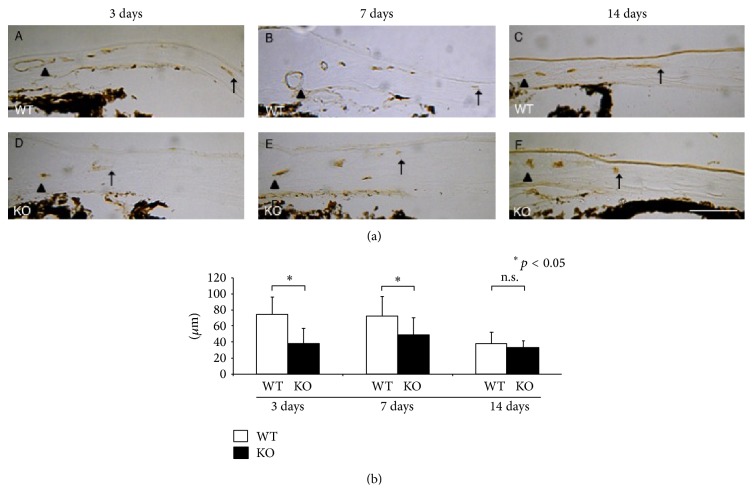
Neovascularization in corneal stroma in histology and evaluation of its length. (a) CD31 immunostaining was performed in cryosections of the mouse cornea. In WT mouse corneas, formation of CD31-labeled neovascularization (arrows) from the limbal vessels (arrowheads) in the corneal stroma was detected in the peripheral cornea at days 3 and 7. The length of the neovascularization was less in KO mice as compared with WT mice at day 3 and day 7 (b). ^∗^ *p* < 0.05; n.s.: not significant. Bar, 100 *μ*m.

We then measured the length of neovascularization in the stroma in histology section. CD31 immunostaining was performed in cryosections of the mouse cornea (Figure 3(a)). In WT mouse corneas, development of the CD31-labeled neovascularization from the limbus in the corneal stroma was detected in the peripheral cornea as early as at day 3 (Figure 3(a)). The length of the neovascularization between the limbus (arrowheads) and the tip (arrows) of new vessels in the corneal stroma was measured at each timepoint.

The length of neovascularization was shorter in KO mice as compared with WT mice at day 3 and day 7, but not at day 14 (Figure 3(b)).

### 3.3. Expression of Inflammatory and Angiogenic Components in Centrally Cauterized Cornea

**Figure 4:**
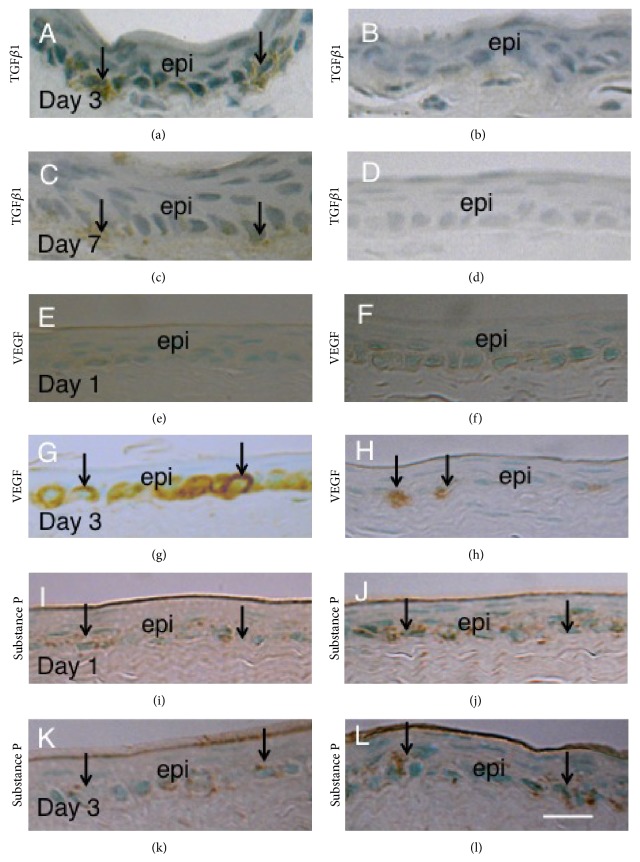
Immunohistochemical detection of angiogenic or inflammatory components in centrally cauterized cornea. Active form of TGF*β*1 was detected in stroma just beneath the epithelium in the area of cauterization at days 3 and 7 in WT mice (arrows in (a) and (c)). Its immunoreactivity was quite less marked in cornea of KO mice ((b) and (d)). At day 1 after cauterization very faint VEGF immunoreactivity was observed in basal cells of corneal epithelia in cauterization area of both WT (e) and KO (f) mice. At day 3 the basal epithelial cell with VEGF immunoreactivity was more frequently observed in a WT cornea (e) as compared with a KO mouse (f). Immunoreactivity for substance P was detected in basal layer of corneal epithelium with no obvious difference in intensity between WT and KO mice at days 1 and 3 ((g)–(l)). Bar, 20 *μ*m.

Immunohistochemistry showed that active forms of TGF*β*1, VEGF, and substance P were not detected in untreated cornea of both genotypes of mice (not shown). Active form of TGF*β*1 was detected in stroma just beneath the epithelium in the area of cauterization at days 3 and 7 in WT mice (Figures 4(a) and 4(c)). Its immunoreactivity was quite less marked in cornea of KO mice (Figures 4(b) and 4(d)). VEGF was also not detected in uninjured corneas of both genotypes of mice. At day 1 after cauterization very faint VEGF immunoreactivity was observed in basal cells of corneal epithelia in cauterization area of both WT and KO mice (Figures 4(e) and 4(f)). At day 3 the basal epithelial cells with VEGF immunoreactivity were more frequently observed in a WT cornea as compared with a KO mouse (Figures 4(g) and 4(h)). Immunoreactivity for substance P was detected in basal layer of corneal epithelium with no obvious difference in intensity between WT and KO mice at days 1 and 3 (Figures 4(i), 4(j), 4(k), and 4(l)).

**Figure 5:**
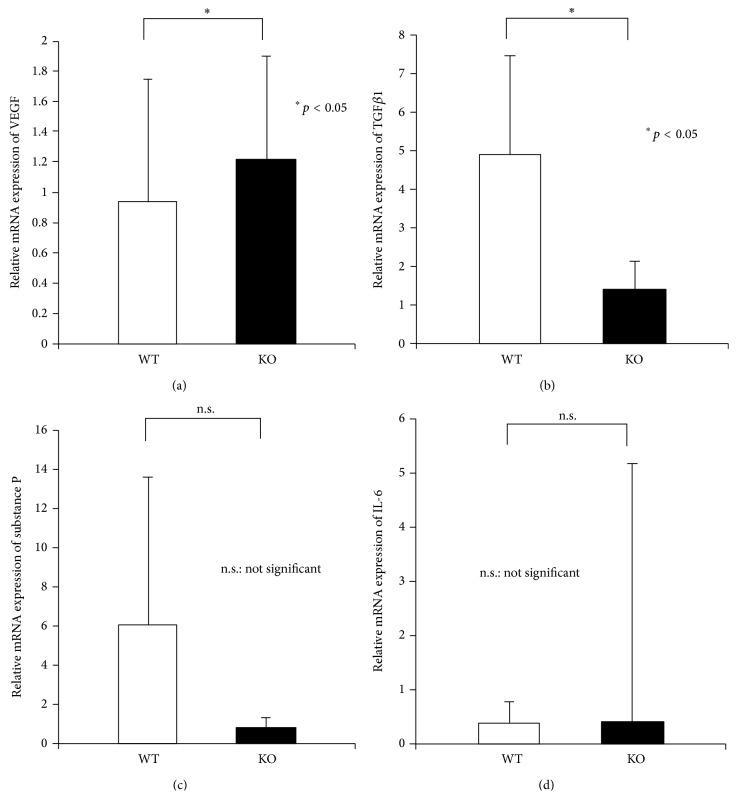
Expression of mRNAs of angiogenic or inflammatory components in centrally cauterized cornea at day 3. mRNA expression of vascular endothelial growth factor (VEGF, (a)) and transforming growth factor *β*1 (TGF*β*1, (b)) mRNAs in the affected cornea of wild-type (WT) was more marked as compared with that in TRPV1-null (KO) mice at day 3 after cauterization. The loss of TRPV1 did not affect mRNA expression level of substance P (c) and IL-6 (d) in the centrally cauterized cornea at this timepoint. ^∗^ *p* < 0.05; n.s.: not significant.

We then examined mRNA expression of VEGF and TGF*β*1, the major two growth factors reportedly involved in corneal neovascularization in day 3 specimens by using real-time RT-PCR. Expression of mRNAs of both VEGF (Figure 5(a)) and TGF*β*1 was significantly less in a KO cornea as compared with a WT cornea at day 3 (Figure 5(b)). TRPV1 signal is reportedly involved in expression of substance P and interleukin-6 (IL-6), both involved in local tissue inflammation. However, in the present study the loss of TRPV1 did not affect mRNA expression level of substance P (Figure 5(c)) and IL-6 (Figure 5(d)) in the centrally cauterized cornea at this timepoint.

### 3.4. Expression of Inflammatory Cell Markers in Centrally Cauterized Cornea

**Figure 6:**
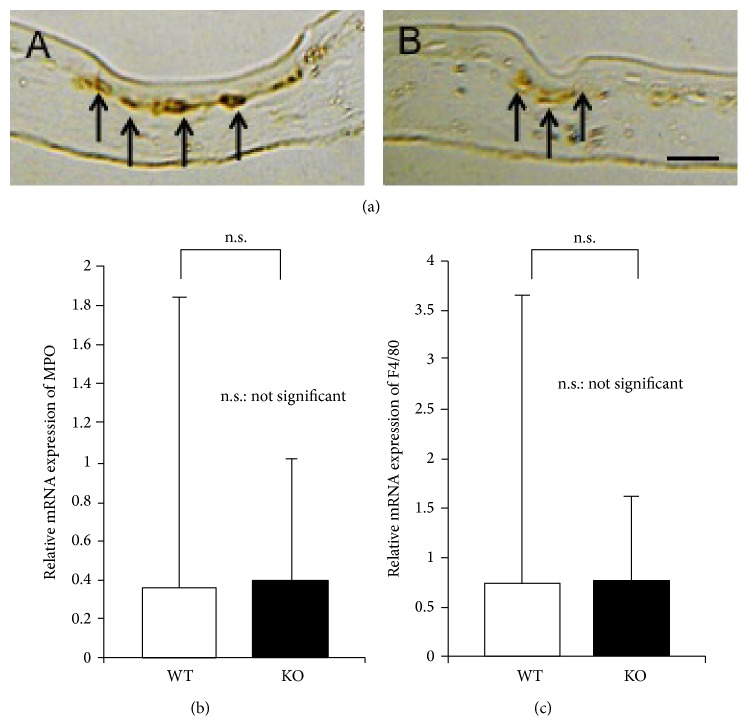
Inflammatory cells in centrally cauterized cornea of WT and KO mice. (a) Immunohistochemistry detected F4/80 labeled macrophages (arrows) beneath the epithelium in the central cornea of both WT (A) and KO (B) corneas at day 7. There is no difference in mRNA expression of myeloperoxidase (MPO), a neutrophil marker (b), and F4/80 macrophage antigen (c) between wild-type (WT) and TRPV1-null (KO) corneas at day 3 after cauterization. n.s.: not significant, bar, 50 *μ*m.

We previously reported that cauterization in the central cornea induced inflammation in the affected area of tissues. We therefore first examined distribution of F4/80-labeled macrophages by using immunohistochemistry and saw difference of distribution of F4/80-labeled macrophages (Figure 6(a)). We then semiquantified the invasion of neutrophils and macrophages in tissues by conducting real-time RT-PCRs for mRNAs of MPO, a neutrophil marker, and F4/80. The loss of TRPV1 exhibited no remarkable effect on mRNA expression of these cell markers (Figures 6(b) and 6(c)).

## 4. Discussion

The present experiments first showed that lacking TRPV1 cation channel receptor suppressed stromal neovascularization in an* in vivo* mouse cornea following receiving a cauterization injury at the central cornea. Neovascularization was found to sprout from the loop-shaped vessels of the limbus toward the center of the corneal stroma in whole-mounted samples of WT tissues, while such neovascularization was much less in a KO cornea at day 3. The distance between limbus and the tip of the neovascularization in the stroma was significantly shorter in a KO cornea and compared with a WT mouse. Cell culture experiment showed that blockage of TRPV1 receptor did not affect VEGF angiogenic action on HUVECs. HUVECs do not reportedly express TRPV1 [26], and thus the present* in vivo* finding of less angiogenesis observed in a KO cornea was not attributed to the direct effects of the loss of TRPV1 on vascular endothelial cells. Although various growth factors that potentially affect neovascularization activity are expressed in an injured cornea during tissue repair [29–32], expression of such components is reportedly modulated by ion channel receptor signaling, that is, signaling derived from TRP family members.

To analyze the mechanism of antiangiogenic effect of lacking TRPV1 in* in vivo* corneal stroma we conducted further experiments. Because TRPV1 signal is involved in inflammation in response to external stimuli, which potentially affect formation of neovascularization, we ran real-time RT-PCR for inflammatory/angiogenic growth factors and inflammation/wound healing-related components. The results showed that mRNA expression of VEGF and TGF*β*1 in cauterized cornea was suppressed by lacking TRPV1, but expression of mRNA of IL-6 was not affected by the loss of TRPV1. Immunohistochemistry also showed that deposition of active form of TGF*β*1 in the stroma beneath the regenerated epithelium and VEGF expression in the basal epithelial cells in the central cornea both seemed less in amount in a KO tissue as compared with a WT mouse. We reported that TGF*β*1 is expressed in corneal epithelium and is deposited in stroma beneath the regenerated epithelium as an active form [33]. Thus, reduced accumulation of active form of TGF*β*1 in the KO cornea might be attributable to the suppression of TGF*β*1 expression in epithelium after cauterization by the loss of TRPV1. Similarly, postcauterization VEGF expression in corneal epithelium was suppressed by lacking TRPV1. IL-6 was not detected by immunohistochemistry presumably because the protein might be secreted out from the cells.

We also examined the level of inflammation in cornea. Immunohistochemistry did not show difference of F4/80-labeled macrophage infiltration. We then ran semiquantification by real-time RT-PCR. The present assessment of inflammatory cell markers, that is, MPO and F4/40, indicated that following cauterization in the central cornea the loss of TRPV1 did not affect infiltration of neutrophil leukocytes and macrophages. Data from these real-time RT-PCR results suggest that less expression of angiogenic growth factors, that is, VEGF and TGF*β*1, is unattributed to the alteration of the level of inflammatory cell infiltration following cauterization in a KO tissue and presumably is dependent on the effects of lacking TRPV1 on the gene expression in resident corneal cells, that is, corneal epithelial cells. Involvement of keratocytes in the suppression of neovascularization in the KO mouse is to be further investigated. TRPV1 in sensory nerve fibers reportedly mediates expression of neuroinflammatory mediators, for example, substance P [34, 35]. In the present study protein and mRNA expressions of substance P were similar in WT and KO corneas after cauterization.

In conclusion, blocking TRPV1 signal might be beneficial in suppression of neovascularization in cornea.
